# Efficacy and safety of Tuina (Chinese Therapeutic Massage) for knee osteoarthritis: A randomized, controlled, and crossover design clinical trial

**DOI:** 10.3389/fmed.2023.997116

**Published:** 2023-04-05

**Authors:** Kaoqiang Liu, Yunfan Zhan, Yujie Zhang, Ye Zhao, Yongli Chai, Hua Lv, Weian Yuan

**Affiliations:** ^1^Department of Orthopedics and Traumatology, Shuguang Hospital, Shanghai University of Traditional Chinese Medicine, Shanghai, China; ^2^Clinical Research Center, Shuguang Hospital, Shanghai University of Traditional Chinese Medicine, Shanghai, China

**Keywords:** Tuina, knee osteoarthritis, clinical trial, crossover design, WOMAC

## Abstract

**Background:**

Knee osteoarthritis (KOA) is a highly prevalent joint disease among the middle-aged and elderly population that can lead to pain, functional impairment, decreased quality of life, and a large number of medical expenses. Physical therapy is one of the main treatment methods for KOA. In China, Tuina has been widely used in the treatment of KOA, but up to now, there is no high-quality medical evidence to support its effectiveness and safety. The purpose of this study was to objectively evaluate the efficacy and safety of Tuina in the treatment of KOA.

**Methods:**

A crossover design clinical trial was performed on 96 patients. The test group and the control group in the trial were allocated randomly in a ratio of 1:1. The test group received Tuina treatment for 4 weeks first and then received health education intervention for another 4 weeks. The control group received health education intervention for 4 weeks first and then received Tuina treatment for another 4 weeks. The Western Ontario and McMaster Universities Arthritis Index (WOMAC) total score was chosen as the primary outcome. The WOMAC pain score, WOMAC stiffness, WOMAC daily activity score, and visual analog scale (VAS) score were the secondary outcomes. Adverse events during the intervention were collected in both groups.

**Results:**

Compared with the baseline, the WOMAC total score, WOMAC pain score, WOMAC stiffness, WOMAC daily activity, and VAS score of patients in both groups were improved significantly at weeks 4 and 8 (*p* < 0.001). All patients who received Tuina treatment were significantly superior to those who received health education intervention in the WOMAC total score (194.96, 95% CI = 164.94–224.97, *P* < 0.001), WOMAC pain score (45.96, 95% CI = 35.82–56.09, *P* < 0.001), WOMAC stiffness (31.42, 95% CI = 26.37–36.46, *P* < 0.001), WOMAC daily activity (117.58, 95% CI = 97.56–137.61, *P* < 0.001), and VAS score (1.07, 95% CI = 0.83–1.32, *P* < 0.001). Both groups had no serious adverse events during the treatment.

**Conclusion:**

This trial demonstrated that Tuina can reduce joint pain in patients with KOA and improve the physical functions of the knee joint effectively and safely.

**Clinical trial registration:**

This trial was registered in the Chinese Clinical Trial Registry (No. ChiCTR-TTRCC-13003157). http://www.chictr.org.cn/showproj.aspx?proj=6402.

## Introduction

Knee osteoarthritis (KOA) is a highly prevalent joint disease among middle-aged and elderly people, and it is a leading cause of pain, impaired function, reduction of life quality, and astonishing medical costs for treatment (1, 2). At the age of 60 years and older, approximately 10% of men and 13% of women have KOA (3). With an aging population and obesity epidemic, the incidence of KOA has been increasing year by year (4). A survey in 2018 found that the overall incidence rate of KOA in China was 18%, 11% in men, and 19% in women (5). American College of Rheumatology (ACR) launched a pyramid scheme of KOA treatment, which was based on measures of education, exercise, and weight reduction, supplementing externally used non-steroidal anti-inflammatory drugs (NSAIDs) if necessary, adding the use of acetaminophen and NSAIDs orally under the ineffective situation, and performing intra-articular corticosteroid injections during acute attack (6). At the same time, definite adverse reactions of NSAIDs have been reported, and the risk of the surgery itself and the high medical cost limited the wide range of clinical applications of the aforementioned therapies. In China, Tuina is one of the main non-surgical treatments for KOA and is included in relevant diagnosis and treatment guidelines (7). Tuina is a manual therapy under the guidance of the meridian theory in traditional Chinese Medicine. It stimulates specific acupuncture points along the pathways of meridians and performs regular passive movements on the patient's joints to alleviate the patient's symptoms. Tuina requires lasting, powerful, uniform, deep, and soft when implemented in clinical treatments. It can be implemented in several ways even at the same point, notably by pressing, pushing, and kneading. Different Tuina treatments should be used for different diseases which have been widely applied in clinical treatments because of their significant efficacy in symptomatic improvement and reduction of pain (8). Many years of clinical practice have shown that Tuina is safe and effective for KOA. Some researchers have also carried out research on its action mechanism on KOA and found that Tuina manipulation and passive movement can regulate the muscle activation mode, repair the function of ligament cartilage, improve the mechanical balance of the knee, improve the inflammatory status, reduce joint pain, and achieve a good therapeutic effect for KOA (9, 10). However, up to now, there has been no high-quality medical evidence to support the effectiveness of Tuina in the treatment of KOA. Although relevant clinical studies have been carried out previously, there are obvious defects in their study design, selection of efficacy indicators, control of treatment confounding factors, quality assurance of clinical trials, etc. Therefore, it is necessary to carry out well-designed and high-quality clinical trials.

## Materials and methods

### Trial design

Considering that KOA is a chronic disease, the symptoms can easily recur, patients with mild knee osteoarthritis (700 ≤ WOMAC total score ≤ 1,200) were included in this study, and the short-term absence of proven effective intervention will not bring obvious risks to patients. To minimize the impact of different patients on the curative effect, this study adopts a randomized, controlled, and crossover design.

**Figure 1:**
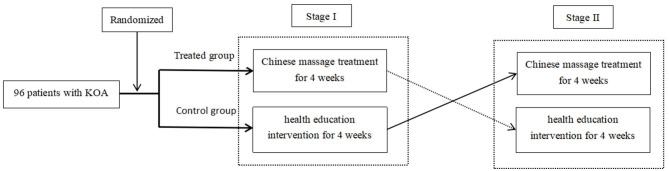
Trial design.

Patients were randomly allocated into the test group and the control group. The test group first received Tuina treatment for 4 weeks and then received health education intervention for another 4 weeks. The control group first received health education intervention for 4 weeks and then received Tuina treatment for another 4 weeks. The Western Ontario and McMaster Universities Arthritis Index (WOMAC) was chosen to evaluate the primary efficacy of the treatment. Referring to the osteoarthritis (OA) treatment cycle and related international clinical trials (CLASS and VIGOR trials), the course of this clinical trial was set for 4 weeks (11, 12). The protocol was approved by the Ethical Review Committee of Chinese Registered Clinical Trials on 15 April 2013 (No. ChiECRCT-2013010) and registered in the Chinese Clinical Trial Registry (No. ChiCTR-TTRCC-13003157). The diagram of the trial design is presented in Figure 1.

### Subjects

A total of 122 patients with KOA were recruited from the osteoarthritis specialist clinic, Shuguang Hospital affiliated with Shanghai University of Traditional Chinese Medicine between July 2013 and August 2016, and 96 patients meeting the criteria were screened into this study.

### Diagnostic criteria

The 2001 American College of Rheumatology (ACR) diagnostic criteria for KOA state that patients who have knee joint pain can be diagnosed with KOA with three of the following seven conditions:

(1) Age ≥ 50 years old.
(2) Morning stiffness < 30 min.
(3) Having bone noises during joint movement.
(4) Examination of the knee shows osseous hypertrophy.
(5) Tenderness and pain of bone.
(6) No obvious temperature rising of synovial membrane.
(7) Radiology shows osteophyte formation.

The inclusion criteria were as follows: subjects who signed written informed consent; age range from 50 to 80 years old (including boundary values); male or female; meeting the diagnostic criteria of KOA; X-ray imaging score of the knee joint; KL classification ≤ 3; the severity of KOA; 700 ≤ WOMAC total score ≤ 1,200; and clinical diagnosis of unilateral knee osteoarthritis.

The exclusion criteria were as follows: subjects with acute fracture damage of the meniscus or surrounding ligament; rheumatic or rheumatoid arthritis; tumor around the knee joint; tuberculosis; idiopathic osteonecrosis of the knee joint; women who are breastfeeding or pregnant; having severe diseases such as cardiovascular, pulmonary, hepatic, renal, and hematopoietic systems, hemophilia, and other hemorrhagic diseases, and mental disease; use of a cardiac pacemaker; received intra-articular injection within 6 months prior to the trial; used disease-improving drugs and cartilage protectors within 6 months prior to the trial; treated with corticosteroids, acupuncture, or physical therapy 1 week before the treatment; the skin of patients on the treatment sites being damaged; long-term use of other drugs that affect the efficacy and safety judgment and have comprehensive treatment; and participated in other studies within 3 months before the trial. The researchers considered these patients inappropriate to be included.

### Sample size calculation

According to the literature reports, the effective rate of Tuina in the treatment of knee osteoarthritis within 4 weeks varies from 75 to 98%. However, we found that the effective rate of Tuina for KOA was far lower than that reported in the literature in clinical practice. Therefore, according to the early clinical practice data, we set the effective rate of Tuina in the treatment of KOA as 55%. It has been reported that ~20% of patients with KOA experienced self-relief within 4 weeks, and the sample size of this study was calculated based on this situation.

Due to the comparison of two sample rates, the sample size was calculated as n = (u_α_+u_β_)^2^/[2(sin^−1^√p_1_-/sin^−1^√p_2_)]^2^, where α = 0.05, u_0.05/2_ = 1.960, β = 0.10, u_0.01_ = 1.282, *p*_1_ = 0.55, and *p*_2_ = 0.2. According to the aforementioned formula, *n* = 40.22 ≈ 40 cases can be obtained. Considering a 20% shedding rate, the sample size of each group is *N* = 48. A total of 96 patients in two groups with KOA were observed.

### Randomization

A total of 96 subjects were selected from those who met the inclusion criteria after screening and randomly allocated into the test group and the control group in a 1:1 ratio.

### Intervention

For the test group, the intervention protocol of Tuina for KOA was formulated according to College Textbooks ***Tuina (Chinese Therapeutic Massage)*** (5th edition). The treatment time was ~15 min, and the frequency was twice a week. To ensure the standardization of the manual treatment scheme, assurance measures have been formulated (see the Quality control section for details). The Tuina treatment protocol for KOA is as Table 1.

**Table 1:** Tuina treatment protocol for KOA.

**Treatment position:**	**The patient lies on their back with relaxed lower limbs. The doctor stands on the affected side**.
Step 1: relaxation manipulation	① ***Kneading:*** Use the left or right palm root or the major thenar eminence to perform a rhythmic spiral movement on the quadriceps femoris from top to bottom. Repeat the manipulation 3–5 times. Do not use too much force. ② ***Grasping:*** The doctor should exert force between the thumb of both hands and the palmar surface of the other four fingers, pinch and lifts the quadriceps femoris and gastrocnemius up and down, and then slowly relax. Repeat the manipulation from the proximal end to the distal end 3–5 times. The strength is based on the patient's tolerance. Do not use too much force. ③ ***Rolling:*** The doctor's right hand should exert force on the dorsal ulnar side, stick it to the quadriceps femoris, and roll back and forth through continuous movement of wrist flexion and extension and forearm pronation and supination. The frequency is about 120 times/min. Repeat the manipulation from top to bottom 3–5 times.
Step 2: Point tapping manipulation	Using their thumb or index finger, the doctor should press the Liangqiu (ST34), Xuehai (SP10), EX-LE2, Dubi (ST35), Yanglingquan (GB34), Zusanli (ST36), and Ashi points around the knee joint slowly for 5–10 s and hook the Weizhong (BL40) and Chengshan (BL 57) with the middle finger or index finger for 5–10 s, taking the patient's feeling of soreness, distension, and pain as the degree of tolerance. Do not exert too much force.
Step 3: Patellar management manipulation	①***Patella lifting:*** Grasp the patella with one hand and five fingers and fix it with the other hand. Lift the patella upward to the maximum extent to make it leave the articular surface of the femoral condyle and repeat the manipulation 3–5 times. ② ***Patella kneading:*** The doctor should press the patella with the palm to perform clockwise or counterclockwise circular kneading and repeat the manipulation 5–10 times.
Step 4: Tendon adjustment manipulation	①***Tendon splitting:*** Press the nail part of the thumb claw against the iliotibial tract, medial collateral ligament, and lateral collateral ligament around the knee joint, and scrape along the fiber direction 3–5 times. ② ***Tendon pulling:*** Place the middle finger pulp of both hands at the inner and outer head of the gastrocnemius muscle and popliteal muscle, respectively, and perform horizontal back and forth pulling 3–5 times.
Step 5: Active joint manipulation	①***Flexion, extension, stretching, and shaking of knee joint:*** The doctor should hold the patient's ankle with one hand and the knee joint with the other hand, flexing and extending the knee joint to the maximum extent 2–3 times, and then quickly pull and shak the knee joint. ② ***Stretching method:*** The doctor should hold the patient's foot with one hand to extend the ankle back and presses the knee with a steady force to the maximum extent that the patient can endure. This should be kept up for 5–10 s.
Step 6: Ending manipulation	***Kneading:*** Use the palm root of the left or right hand or thenar part to exert force, and perform rhythmic spiral movement from top to bottom on the quadriceps femoris and tibialis anterior muscle of the patient. Repeat the manipulation 3–5 times. Do not exert too much force.

For the control group, patients were given health education in the prescribed research period, including knowledge publicity and education related to KOA for patients, teaching patients to correct unhealthy living habits, keeping knee joints warm, and avoiding sports that would aggravate injury of the knee joint. At the same time, patients were told not to receive other treatments of KOA during the observation period. To ensure the quality of health education, patients were told to visit the hospital clinic at least once a week. During the study, the subjects can consult the research doctor about relevant health problems by telephone at any time.

Combined treatment and medication regulations: during the period of trial, except for the regulated intervention methods, no other Chinese or Western drugs for osteoarthritis and no other treatment methods are allowed. The medical case should be recorded as ineffective if analgesic drugs are used for KOA. The name and dosage of the drug used should be recorded clearly.

### Outcome measurements

#### Primary outcome—WOMAC total score

The Western Ontario and McMaster Universities Arthritis Index (WOMAC) is mainly used to evaluate patients' overall evaluation of their disease in the past 48 h. The specific content of the scale includes 24 questions from three aspects of pain, stiffness, and difficulty of daily activities (the maximum score of each question is 100, and the total score is 2,400):

- **Pain score :** It contains five questions—the degree of pain when walking on a flat road, the degree of pain when going upstairs or downstairs, the influence of knee joint pain on nocturnal sleep, the degree of pain when sitting or lying down, and the degree of pain when standing upright (the maximum score of each question is 100, and the total pain score is 500).
- **Stiffness:** It contains two questions. How severe is your stiffness when you just wake up in the morning? How severe is your stiffness after sitting, lying, or resting? (the maximum score of each question is 100, and the total stiffness score is 200).
- **Difficulty in daily activities:** It contains 17 questions. Going downstairs; going upstairs; standing up from sitting; standing; walking on a flat ground; getting on and off the car or bus; going shopping; wearing ones socks or stockings; standing up from bed; drawing off ones socks or stockings; lying on the bed; getting out of the bathtub; sitting; sitting on, or standing up from toilet; doing heavy homework; and doing easy homework (the maximum score of each question is 100, and the total score of daily activities is 1,700).

#### Secondary outcome—Visual analog scale score

The visual analog scale (VAS) score is a national general pain evaluation index, and this method uses a 10 cm line or ruler. There are 0 and 10 at each end; 0 means painless, and 10 means the most severe pain. The patient was told to mark the corresponding position of their pain on a straight line or ruler.

### Safety

Adverse events during the intervention were collected in both groups.

### Statistical analysis

The statistical software was SPSS21.0. The chi-square test was used to compare and analyze differences between count data. For numerical data subject to a normal distribution, an independent sample *t*-test was used to assess the inter-group difference, and paired sample *t*-test was used to assess the intra-group difference. For numerical data not subject to the normal distribution, the Mann–Whitney *U*-test was used to assess the inter-group difference, and the Wilcoxon signed rank-sum test was used to assess the intra-group difference. The ANOVA test for two-stage crossover design was used to analyze the differences in efficacy by different intervention factors, stage, and individual differences.

All numerical data were expressed by mean ± standard deviation (M ± SD). All statistical tests used a two-sided test, and a *P*-value of < 0.05 is considered statistically significant. Patients who took other drugs for osteoarthritis were regarded as having no efficacy. Intention-to-treat analysis (ITT) was used to evaluate the efficacy. For the estimation of the missing value of the main efficacy outcome, the results of the latest observation were carried forward to the missing part of the trial data.

### Quality control

(1) Overall quality control measures. The overall quality of this clinical study was implemented with reference to good clinical practice (GCP), and the protection of subjects followed the Declaration of Helsinki. The research group hired a full-time monitor for quality supervision. (2) Quality control measures for consistency of Tuina manipulation. Qualification requirements were as follows: having practiced manual therapy for at least 5 years. Access requirements were as follows: key parameters of manual operation should be formulated. Before the formal start, the research group shall carry out manipulation training for all research doctors. Relevant training, examination videos, and written records should be well-kept. Process quality control: a standardized spot check should be regularly and irregularly conducted on manipulative doctors during the process. (3) Efficacy index quality control. Since the blind method cannot be implemented in this study, to ensure the reliability of the research results, an independent efficacy evaluation and safety event judgment researcher were set up for this study. The researcher does not participate in the subject intervention and does not know what kind of intervention the subject has received.

## Results

**Figure 2:**
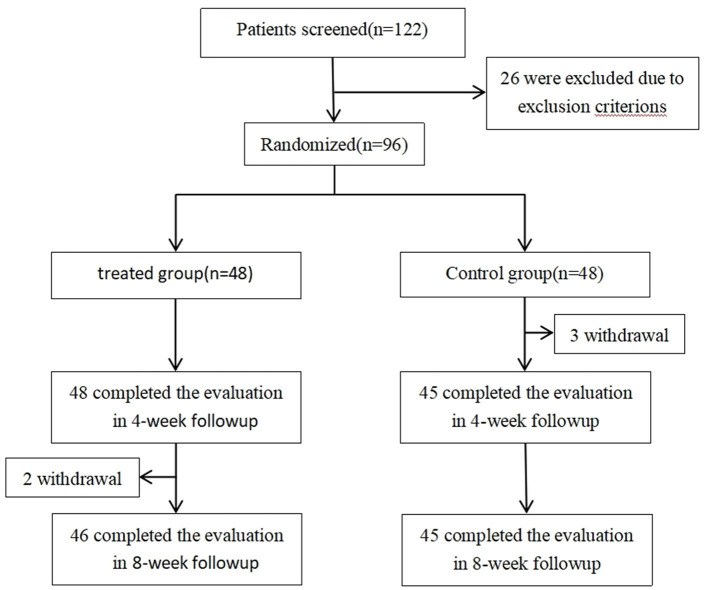
Flow diagram of participant screening and randomization.

A total of 96 patients were randomized into a test group and a control group, 48 patients in each group. Two patients in the test group withdrew in the 2nd week of the second stage, and three patients in the control group, respectively, withdrew in the 2nd, 3rd, and 4th week of the first stage (Figure 2; flow diagram of participant screening and randomization for details). Two groups are comparable in age, gender, BMI, X-ray image classification, WOMAC total score, WOMAC pain score, WOMAC stiffness score, WOMAC daily activity score, and VAS score (detailed baseline data of 96 patients are shown in Table 2).

**Table 2:** Comparison of baseline data between the two groups (M ± SD).

**Observation index**	**Test group (*N* = 48)**	**Control group (*N* = 48)**	***p***
Gender (*n*, %)	Female (30, 62.5%)	Female (28, 58.3%)	0.676
diseased knee joint (*n*, %)	Right (37, 77.1%)	Right (33, 68.8%)	0.358
X-ray KL	I18/II25/III5	I21/II24/III3	0.687
Age, year	60.33 ± 3.44	59.56 ± 3.57	0.394
BMI	25.20 ± 2.83	25.77 ± 2.34	0.284
WOMAC-total score	791.02 ± 146.14	773.44 ± 82.34	0.750
WOMAC-pain score	188.71 ± 33.68	183.92 ± 18.52	0.803
WOMAC-stiffness score	97.29 ± 15.90	94.60 ± 13.33	0.372
WOMAC-daily activity score	505.02 ± 111.17	494.92 ± 66.05	0.585
VAS-score	4.94 ± 1.08	4.79 ± 1.05	0.579

M ± SD, mean ± standard deviation; X-ray KL, X-ray Kellgren-Lawrence; BMI, body mass index; WOMAC, Western Ontario and McMaster Universities Arthritis Index; VAS, visual analog scale.

The analysis of variance (ANOVA) test for two-stage crossover designs was used to analyze the differences among intervention effects, stage effects, and individual differences. The results have shown that both the intervention effect and the stage effect contributed to the difference in the WOMAC total score, WOMAC pain score, WOMAC stiffness score, WOMAC daily activity score, and VAS score (*p* < 0.05). Detailed results are shown in Table 3. However, as shown in Table 4, the change value of the second stage is smaller than that of the first stage, so the stage effect reduces the difference between the two treatments. In this case, the difference between the two treatments is still statistically significant, indicating that the conclusion is still reliable.

**Table 3:** ANOVA test for two-stage crossover effects.

**Observation index**	**Source of variance**	***F***	***P*-value**
WOMAC-total score	Intervention effect	132.539	< 0.001
	Stage effect	24.034	< 0.001
	Individual difference	0.614	0.991
WOMAC-pain score	Intervention effect	60.421	< 0.001
	Stage effect	46.009	< 0.001
	Individual difference	0.505	0.999
WOMAC-stiffness score	Intervention effect	121.713	< 0.001
	Stage effect	8.099	0.005
	Individual difference	0.606	0.992
WOMAC-daily activity score	Intervention effect	112.264	< 0.001
	Stage effect	9.841	0.002
	Individual difference	0.678	0.970
VAS-score	Intervention effect	67.794	< 0.001
	Stage effect	17.950	< 0.001
	Individual difference	0.902	0.691

WOMAC, Western Ontario and McMaster Universities Arthritis Index; VAS, visual analog scale.

**Table 4:** Outcome comparison between the two groups.

**Observing index**	**group**	***N***	**0 W (M ±SD)**	**4 W (M ±SD)**	**8 W (M ±SD)**	**d1 (M ±SD) (95% CI)**	**d2 (M ±SD) (95% CI)**	*P*-value^*^
WOMAC-total score	Test group	48	791.02 ± 146.14	423.52 ± 192.62	334.00 ± 207.78	367.5 ± 120.92 (−402.61 to −332.39)^‡^	89.52 ± 88.58 (−115.24 to −63.80)	< 0.001
	Control Group	48	773.44 ± 82.34	588.35 ± 129.56	291.33 ± 76.08	185.08 ± 85.9 (−210.03 to −160.14)	297.02 ± 120.83 (−332.11 to −261.93)	< 0.001
WOMAC-pain score	Test group	48	188.71 ± 33.68	83.69 ± 46.09	64.73 ± 50.15	105.02 ± 33.65 (−114.79 to 95.25)^‡^	18.96 ± 29.32 (−27.47 to −10.45)	< 0.001
	Control Group	48	183.92 ± 18.52	124.13 ± 39.71	58.48 ± 28.67	59.79 ± 35.49 (−70.10 to −49.49)	65.65 ± 42.63 (−78.02 to −53.27)	< 0.001
WOMAC-stiffness score	Test group	48	97.29 ± 15.90	47.63 ± 21.24	37.48 ± 22.01	49.67 ± 17.52 (−54.75 to 44.58)^‡^	10.15 ± 15.69 (−14.70 to −5.59)	< 0.001
	Control Group	48	94.60 ± 13.33	76.08 ± 21.89	34.25 ± 15.63	18.52 ± 17.46 (−23.59 to −13.45)	41.83 ± 19.93 (−47.62 to −36.05)	< 0.001
WOMAC-daily activity score	Test group	48	505.02 ± 111.17	292.21 ± 132.41	231.79 ± 143.29	212.81 ± 84.72 (−237.41 to −188.21)^‡^	60.42 ± 57.22 (−77.03 to −43.80)	< 0.001
	Control Group	48	494.92 ± 66.05	388.15 ± 83.19	198.60 ± 46.62	106.77 ± 55.98 (−123.02 to −90.52)	189.54 ± 78.75 (−212.41 to −166.67)	< 0.001
VAS-score	Test group	48	4.94 ± 1.08	2.56 ± 1.20	1.81 ± 1.28	2.38 ± 0.98 (−2.66 to −2.09)^‡^	0.75 ± 0.70 (−0.95 to −0.55)	< 0.001
	Control Group	48	4.79 ± 1.05	3.17 ± 1.06	1.02 ± 0.67	1.63 ± 0.61 (−1.80 to −1.45)	2.15 ± 1.09 (−2.46 to −1.83)	< 0.001

d1, difference between 0 and 4 w (95% CI).

d2, Difference between 4 and 8 w (95% CI).

^*^Significant difference from 4-week group vs. baseline and 8-week group vs. 4 weeks.

^‡^Significant difference from the control group.

M ± SD, mean ± standard deviation; WOMAC, Western Ontario and McMaster Universities Arthritis Index; VAS, visual analog scale.

Compared with the baseline, the WOMAC total score, WOMAC pain score, WOMAC stiffness, WOMAC daily activity, and VAS score of patients in both groups improved significantly at weeks 4 and 8 (*p* < 0.001). However, at week 4, the test group was superior to the control group in the WOMAC total score, WOMAC pain score, WOMAC stiffness, WOMAC daily activity, and VAS score (*p* < 0.001). Detailed results are shown in Table 4.

**Figure 3:**
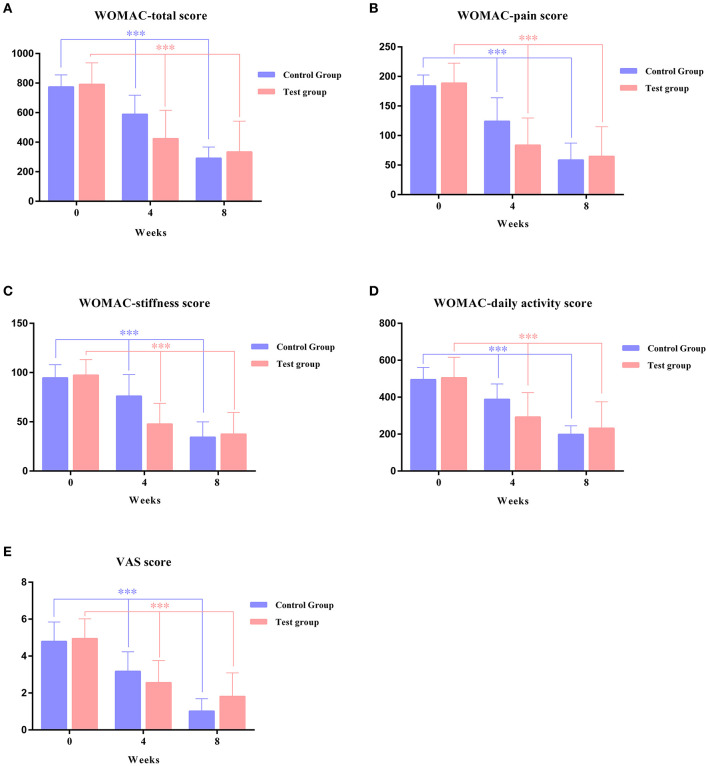
Changes in primary outcomes over an 8-week study period between the test group and control group. **(A)** WOMAC total score, **(B)** WOMAC pain score, **(C)** WOMAC stiffness score, **(D)** WOMAC daily activity score, and **(E)** VAS score. ***Significant difference from 4-week group vs. baseline and 8-week group vs. 4 weeks.

At the same time, an independent sample *t*-test was used to compare and analyze differences between different intervention effects (Tuina and health education). The results showed that the mean difference between the efficacy of the Tuina treatment and health education was significant in the WOMAC total score (194.96, 95% CI = 164.94–224.97, *P* < 0.001), WOMAC pain score (45.96, 95% CI = 35.82–56.09, *P* < 0.001), WOMAC stiffness (31.42, 95% CI = 26.37–36.46, *P* < 0.001), WOMAC daily activity (117.58, 95% CI = 97.56–137.61, *P* < 0.001), and VAS score (1.07, 95% CI = 0.83–1.32, *P* < 0.001). Tuina therapy was superior to health education in improving the WOMAC total score, WOMAC pain score, WOMAC stiffness, WOMAC daily activity, and VAS score (*p* < 0.001). Detailed results are shown in Tables 4, 5 and Figure 3.

**Table 5:** Comparison of change in different intervention factors.

**Observing index**	*N* ^*^	**Tuina treatment^‡^**	**Health education^†^**	**Estimated difference (95% CI)**	***t***	***P*-value**
WOMAC-total score	96	332.26 ± 125.35	137.30 ± 99.19	194.96 (164.94–224.97)	11.95	< 0.001
WOMAC-pain score	96	85.33 ± 43.02	39.38 ± 38.34	45.96 (35.82–56.09)	7.81	< 0.001
WOMAC-stiffness	96	45.75 ± 19.08	14.33 ± 17.04	31.42 (26.37–36.46)	12.04	< 0.001
WOMAC-daily activity	96	201.18 ± 82.19	83.59 ± 60.93	117.58 (97.56–137.61)	11.26	< 0.001
VAS-score	96	2.26 ± 1.04	1.19 ± 0.79	1.07 (0.83–1.32)	8.07	< 0.001

^*^The total number of patients receiving the same treatment.

^‡^The change value of efficacy indexes of all patients receiving Tuina treatment in both groups.

^†^The change value of efficacy indexes of all patients receiving health education in both groups.

WOMAC, Western Ontario and McMaster Universities Arthritis Index; VAS, visual analog scale.

### Safety

Both groups had no serious adverse events during the treatment. A total of two female patients in the test group had local skin pain around the knee joint after Tuina therapy, which was caused by the stimulation of manipulation, and completed all treatments without special treatment. One patient in the control group withdrew because the pain in the knee joint increased during health education. A total of two patients in the control group took celecoxib (0.2 g, qd, po) because of increased pain during health education.

## Discussion

Tuina is a manual therapy under the guidance of the meridian theory of traditional Chinese Medicine. It stimulates specific acupuncture points along the pathways of meridians and performs regular passive movements on patients' joints to alleviate patients' symptoms. As for studies on its action mechanism for knee osteoarthritis, Salter (13, 14) confirmed that continuous passive exercise during an early period can help repair and regenerate cartilage through long-term research. It is believed that repeated joint flexion and extension exercise can stimulate the transformation of undifferentiated interstitial cells in cartilage tissue into cartilage cells and accelerate the repair of cartilage tissue. When applied to the affected site of patients with KOA, mechanical receptors on the surface of articular cartilage cells can transform mechanical signal into a chemical signal, thus regulating the proliferation and differentiation of articular cartilage cells, accelerating the repair of articular cartilage, and relieving articular cartilage degeneration (15). The study of Chen et al. (16) has shown that Tuina can relieve the degeneration of knee cartilage in patients with KOA. However, animal experiments (17) confirmed that Tuina can help to prevent KOA; however, it cannot completely stop the process of articular cartilage degeneration. In addition, its effect was different from that of the sodium hyaluronate group. The experiment results of Dai et al. (18) showed that the content of 8-hydroxydeoxyguanosine in the Tuina group was lower than that in the control group. It might suggest that the Tuina treatment had certain efficacy in reducing damage to the DNA of cartilage cells by oxygen-free radicals. In terms of hemodynamics, previous studies (19, 20) have shown that its action mechanism might be related to the passive activity of the joint, which can promote the infiltration and diffusion of synovia to articular cartilage, improve nutritional metabolism of tissue, benefit blood circulation around the joint, reduce intraosseous pressure, and accelerate self-repair of tissue around the joint. In morphology, Tang and Du (21) applied kneading manipulation to both sides of the patella, knee joint, and the posterior fossa of the knee joint to modeled Wister rats, followed by bending and stretching the knee joint. The results were that the degenerative changes of articular cartilage in the manipulation group and the sodium hyaluronate group, such as vacuolar degeneration of cytoplasm, decreased quantity, and degraded function of articular chondrocytes, nucleus pyknosis, and necrosis even disintegration of cells, were lesser than that in the group without treatment.

This study shows that Tuina has significant efficacy on stiffness and activities of daily living. The action mechanism may be related to its functions in regulating muscle balance and improving the abnormal biomechanical state of the knee joint. Limitation range of motion of the knee joint in patients with KOA is closely associated with fatigue of the quadriceps femoris (22). Tuina manipulation helps to stretch muscle fibers, improve muscle tension, endurance, and elasticity, and correct biomechanical disorder of the knee joint. Therefore, it can speed up the recovery of knee muscle strength and improve its stability (23). Gong et al. (24) treated patients with KOA with Tuina and found that quadriceps femoris peak torque (PT), single total work (TW), and average power (AP) were all significantly increased by the isokinetic muscle strength test. The Lequene and Mery severity index and the JOA score were also improved significantly.

A 4-week Tuina therapy can significantly improve clinical symptoms in patients with KOA. This study also confirms that Tuina can reduce pain in patients with KOA effectively. The mechanism may be related to its functions in improving local blood circulation and reducing levels of inflammatory cytokines. Repeated rubbing stimulation to the affected knee can increase the temperature in the deep tissue, dilate blood vessels, accelerate the blood flow rate of the knee joint, improve local microcirculation, and thus promote absorption of inflammatory substances. Meanwhile, it can also promote synovial fluid secretion and improve cartilage nutrition. Best et al. (25) believed that absorption may increase tissue revascularization by upregulating VEGF. Previous studies (26–29) have shown that absorption can regulate synovial blood flow of the knee joint in patients with KOA, inhibit the release of PGE2, reduce levels of IL-1, IL-6, TNF-α, MMP-3, relieve pain, protect the knee joint, increase levels of serum osteoprotegerin (OPG) and osteocalcin (BGP), and promote osteogenesis.

Meanwhile, there are limitations of this study, such as the follow-up time being too short to objectively evaluate the long-term efficacy of Tuina for KOA. At the same time, the blind method cannot be realized due to the large differences in intervention measures, and the primary and secondary outcomes were non-objective indicators, bringing great challenges to research quality assurance. However, to maximize the reliability of the result, our study group took some measures in the process. For example, we hired a full-time monitor for quality supervision, developed quality control measures for consistency of Tuina manipulation, and set up an independent efficacy evaluation and safety event judgment researcher in the study.

## Conclusion

This is a crossover, randomized, and controlled clinical trial to provide evidence that Tuina therapy is safe and effective for patients with KOA. It demonstrated that a 4-week Tuina therapy period can effectively and safely increase physical functions of the knee joint, reduce joint pain, and improve the quality of life in patients with KOA compared with health education.

## Data availability statement

The original contributions presented in the study are included in the article/supplementary material, further inquiries can be directed to the corresponding author.

## Ethics statement

The studies involving human participants were reviewed and approved by Chinese Ethics Committee of Registering Clinical Trials. The patients/participants provided their written informed consent to participate in this study.

## Author contributions

WY designed this trial, led this trial quality assurance, and revised the manuscript. KL and YZhan drafted and revised this manuscript. HL contributed to data statistical analysis. YZhang, YZhao, and YC performed data interpretation. All authors have read and approved the final manuscript.
